# Supplementary material for: On the Biosynthesis of Bioactive Tryptamines in Black Cohosh (Actaea racemosa L.)
Source: Plants (Basel). 2025 Jan 20;14(2):292. doi: 10.3390/plants14020292 (PMC11768127; doi:10.3390/plants14020292)
Supplement: Supplementary file 1 [file plants-14-00292-s001.zip › plants-3408171-supplementary.pdf]

Supplementary Table S1. Primers used in this study.

Name	Sequence (5′ → 3′)
CPR1-pYES-KpnI-5	CCTCCTGACGGTACCACACAATGTCCCAATCTGAATCTGTCAAGTTCTC
CPR1-pYES-XbaI-3	GCAGCCGACCTCTAGATCACCACACGTCACGCAGATACCT
Ble5'F	GAGACCTTCGTTTGTGCGGATCC
Ble5'Fnest+AscI	GTACTTGGCGCGCCCCCACACACCATAGCTTCAAAATGT
Ble3'R+URA3term	GTATACATGCATTTACTTATAATACAGTTTTTCAGTCCTGCTCCTCGGCCAC GA
EF-1aF	GAAGCTGGTATCTCCAAGGATG
EF-1aR	GGAATCTTGTCTGGGTTGTACC
pYES2F	GATGAATTGAATTGAAAAGCTAGCTTATCG
pYES2Fnest+AscI	GTACTTGGCGCGCCGCTTATCGATGATAAGCTGTCAAAGATGAG
pYES2KpnOsT5Hfwd	AAGTACTAGGTACCACACAATGTCCCTCACCATGGCGTCGACGATG
pYES2XbaOsT5Hrev	AAGACACGTCTAGACCTCACTAAGCTCCTCTCCCTT
OsT5H_N-termR33_T5H-L1 3'	GAAGCCTAGTTGGACCAGGAGGGAGCCGCCGTGGCTTTGACGACG
RiceT5Hfwd2splice	GACAATCTCAAGGCCCTCGTCCTGGACATGTTCGTCGCCGGCACGGA
RiceT5Hrev2splice	TCCGTGCCGGCGACGAACATGTCCAGGACGAGGGCCTTGAGATTGTC
T5H-L2-pYES-KpnI-5	CCTCCTGACGGTACCACACAATGTCCTTCCAATGGGTAGAACAGTGTGG
T5H-L2-pYES-XbaI-3	GCAGCCGACCTCTAGACAGAGCTGCGCAGAGTTGCAGC
T5H-L1-pYES-KpnI-5	CCTCCTGACGGTACCACACAATGTCCGATATCTACTTGCTCCATGAAC

T5H-L1-pYES-XbaI-3	GCAGCCGACCTCTAGACCAGATTGGCGCTTCTGGCTATTAAG
T5H-L1F2intronsplice	GATCAAATCAAGGGAGTCCTCACGGACATGTTCCTTGCTGGAACTGATAC
T5H-L1R2intronsplice	GTATCAGTTCCAGCAAGGAACATGTCCGTGAGGACTCCCTTGATTTGATC
T5H-L1F_OsT5H_N-termR33	CGTCGTCAAAGCCACGGCGGCTCCCTCCTGGTCCAACTAGGCTTC
T5H-L1trunc-pYES-KpnI-5	CCTCCTGACGGTACCACACAATGTCCGCATTAGGTATGAGAAAGCTCCCT
T5H-L2trunc-pYES-KpnI-5	CCTCCTGACGGTACCACACAATGTCCCGAACTAGCAAACTGAACTTACC
T5HdegF2	GCTGGATTCTGCATCGGAGAYTTYTTYCC
T5HdegR2	GAAGGTAGTATCAGTTCCAGCCACRAACATRTC
TDC PLPThr fwd	ACTTGGATTGTACTTGTTTATGGGTTAAAC
TDC PLPThr rev	GTTTAACCCATAAACAAGTACAATCCAAGT
TDC-PET-5-NcoI	CGGTAACGGCCATGGGTAGTCTCCCTGCCAAC
TDC1 PLPC325S fwd	ACTTGGATTGTTCTTGTTTATGGGTTAAAC
TDC1 PLPC325S rev	GTTTAACCCATAAACAAGAACAATCCAAGT
TDC1 PLPK316R fwd	AGTCCACATAGATGGCTTTTAACTTACTTGG
TDC1 PLPK316R rev	CCAAGTAAGTTAAAAGCCATCTATGTGGACT
TDC1-pYES-Kpn-5	CCTCCTGACGGTACCACACAATGTCCAGTCTCCCTGCCAACATA
TDC2 PLPK314R fwd	AGTCCGCATAGATGGCTTTTAACTTACTTGG
TDC2 PLPK314R rev	CCAAGTAAGTTAAAAGCCATCTATGCGGACT
TDC2 PLPS323C fwd	ACTTGGATTGTTGTTGTTTATGGGTTAAAC
TDC2 PLPS323C rev	GTTTAACCCATAAACAACAACAATCCAAGT
TDC-3-HindIII	GTCGCTGAGAAGCTTATTCATCATTCTCCCACCAACATATC

TDC2-pYES-Kpn-5	CCTCCTGACGGTACCACACAATGTCCGGTAGTTTCCCTGCCAACAT
TDC-pYES-Xba-3	GCAGCCGACCTCTAGACTTCTCCCACCAACATATCAGC
TDCqPCRF	TGTTCGAAGGGTTCGTCAAATCGG
TDCqPCRR	ACGTACGTCCCTCCTATCACTGTA
URA3term+Ble3'	TCGTGGCCGAGGAGCAGGACTGAAAAACTGTATTATAAGTAAATGCATGTATAC

Underlined bases represent cutting sites for restriction enzymes given in the primer names.
